# Malaria in three epidemiological strata in Mauritania

**DOI:** 10.1186/s12936-016-1244-3

**Published:** 2016-04-12

**Authors:** Mohamed Ouldabdallahi Moukah, Ousmane Ba, Hampaté Ba, Mohamed Lemine Ould Khairy, Ousmane Faye, Hervé Bogreau, Frédéric Simard, Leonardo K. Basco

**Affiliations:** Initiative mauritanienne pour la lutte contre les maladies endémiques « MEDCINGO » , ilôt 358, Riyad Pk8, Nouakchott, Mauritania; Laboratoire de parasitologie-mycologie, Institut National de Recherches en Santé Publique (INRSP), B.P. 695, Nouakchott, Mauritania; Programme National de Lutte contre le Paludisme (PNLP), Ministère de la Santé, Nouakchott, Mauritania; Laboratoire d’Ecologie Vectorielle et Parasitaire, Faculté des Sciences et Techniques, Université Cheikh Anta Diop (UCAD), Dakar, Senegal; Unité de Recherche sur les Maladies Infectieuses et Tropicales Emergentes (URMITE), Faculté de Médecine La Timone, Institut de Recherche pour le Développement (IRD) 198, Aix-Marseille Université, Marseille, France; Unité de Parasitologie et d’Entomologie, Département des Maladies Infectieuses, Institut de Recherche Biomédicale des Armées (IRBA), Brétigny-sur-Orge, France; Laboratoire de Parasitologie, Centre National de Référence du Paludisme région Antilles-Guyane, Institut Pasteur de la Guyane, 23 avenue Pasteur, BP 6010, 97306 Cayenne cedex, France; Unité Mixte de Recherche MIVEGEC « Maladies Infectieuses et Vecteurs: Ecologie, Génétique, Evolution et Contrôle » , Institut de Recherche pour le Développement (IRD) 224-Centre National de la Recherche Scientifique 5290, Université de Montpellier (UM), 911 avenue Agropolis, 34394 Montpellier cedex 5, France

**Keywords:** Epidemiology, Prevalence, *Plasmodium*, *Anopheles*, Drug resistance, Mauritania

## Abstract

**Background:**

Malaria epidemiology in Mauritania has been characterized on the basis of epidemiological strata, defined by climatic and geographic features, which divide the country into three zones: Sahelian zone, Sahelo-Saharan transition zone, and Saharan zone. The association between geographic stratification and malaria transmission was assessed through a series of parasitological and entomological surveys.

**Methods:**

Surveys were conducted during the ‘cool’ dry season in 2011, ‘hot’ dry season in 2012, and rainy season in 2013 in a total of 12 sentinel sites. Finger-prick capillary blood samples were collected from children aged 2–9 years old in randomly selected households for microscopic examination and rapid diagnostic test for malaria. Adult mosquitoes were sampled by pyrethrum spray catch and CDC light traps and identified using morphological keys and molecular tools.

**Results:**

Of 3445 children included, 143 (4.15 %) were infected with malaria parasites including *Plasmodium falciparum* (n = 71, 2.06 %), *Plasmodium vivax* (57, 1.65 %), *P. falciparum*-*P. vivax* (2, 0.06 %), *Plasmodium ovale* (12, 0.35 %), and *Plasmodium malariae* (1, 0.03 %). A large majority of *P. falciparum* infections were observed in the Sahelo-Saharan zone. Malaria prevalence (*P* < 0.01) and parasite density (*P* < 0.001) were higher during the rainy season (2013), compared to cool dry season (2011). *Plasmodium vivax* was mainly observed in the Saharan region [43 of 59 (73 %) *P. vivax* infections], mostly in Nouakchott districts, with no significant seasonal variation. Of 3577 mosquitoes captured, 1014 (28.3 %) belonged to *Anopheles* spp. *Anopheles gambiae* was the predominant species in all three epidemiological strata during the ‘cool’ dry season in 2011 but was absent in all study sites, except for Teyarett district in Nouakchott, during the ‘hot’ dry season in 2012. During the rainy season in 2013, *An. gambiae*, *Anopheles arabiensis*, *Anopheles pharoensis,* and *Anopheles rufipes* were abundant in different zones.

**Conclusions:**

The results of the present study support the stratification of malaria in Mauritania. However, the Sahelian zone had the lowest malaria prevalence, while the Sahelo-Saharan zone had the highest malaria burden. Local changes due to anthropogenic factors (i.e., human migration, urbanization, malaria interventions) should be considered in order to optimize the control strategy.

## Background

The current malaria situation in Mauritania is not well known. Although public health centres and hospitals, mostly situated in the eastern and southern regions, report an average of 200,000 malaria cases every year, a large majority of these cases are based on presumptive clinical diagnosis [1]. According to the official government documents, malaria accounts for 25 % of morbidity and 39 % of mortality in health centres located in the Sahelian zone where *Plasmodium falciparum* is endemic [1]. However, there are many areas in the country where malaria surveys, including ‘malaria indicator survey’, have not been conducted using reliable diagnostic tools. The existing data on malaria epidemiology are scarce and have not been updated, with the exception of Nouakchott, the capital city of Mauritania [2–8]. The current knowledge, or lack thereof, on malaria epidemiology in Mauritania has been reviewed recently [9].

**Fig. 1:**
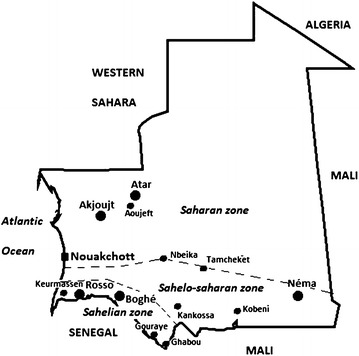
Study sites of the present study. Three epidemiological strata are delineated. In addition to 12 study sites of the present study, the location of other cities and towns (Atar, Nema, and Rosso) mentioned in the text is indicated in the figure

Mauritania can be divided into three major epidemiological strata based on climatic and geographic features: (i) Sahelian zone (annual rainfall, 300–500 mm) along the Senegal River where artificially irrigated rice fields and dams are widespread and malaria transmission is thought to be perennial; (ii) Sahelo-Saharan transition zone (annual rainfall, 100–300 mm) in the southeastern region of the country, south of an artificial line drawn from Nouakchott to Nema (see Fig. 1), where the majority of the population practices seasonal rain-dependent agriculture and semi-nomadic livestock breeding and malaria transmission is seasonal and thought to last up to six months; and (iii) the Sahara desert (annual rainfall, 50–100 mm) to the north of the Nouakchott-Nema axis where malaria transmission is low and thought to occur episodically in oases [1, 9]. The direct association between the geographic stratification and malaria transmission has not been validated through field studies.

As Mauritania attempts to move forward with an ambitious goal to reduce malaria-associated morbidity and mortality by 75 % before 2015 and eventually eliminate malaria from the country [1], there is an urgent need to establish an evidence-based malaria prevalence assessment in the general population living in different epidemiological strata that purportedly characterize malaria transmission pattern in the country. The present study was conducted with the aim to collect and analyse recent and reliable parasitological and entomological data from 12 study sites located in three different epidemiological strata during three time periods: during the ‘cool’ dry season after the rainy season (November–December 2011), during the ‘hot’ dry season (June 2012), and during the rainy season (October 2013). The target population consisted of children aged 2–9 years old.

## Methods

### Study sites

This study was conducted in a total of 12 sentinel sites: four in the Sahelian zone (Keurmassen, Boghé, Gouraye, and Ghabou), four in Sahelo-Saharan transition zone (Kobeni, Tamcheket, Kankossa, and Nbeika), and four in the Saharan zone (Aoujeft, Akjoujt, and two districts in Nouakchott, the capital city, Teyarett and Darnaim) (Fig. 1). Their distinctive geographic and climatic features are described in Table 1 [10]. The selection of these sites was based primarily on the mean annual rainfall and either the presence of agricultural activities or a relatively large body of water or proximity to the Senegal River (for sites in the Sahelian zone), and secondarily on their accessibility, the presence of a public health centre, and the level of community participation.

**Table 1:** Study sites and their characteristics

Sites	Province, region^a^	Location	Number of inhabitants^b^	Key geographic and climatic features
Sahelian zone				
Keurmassen	Keurmassen, Trarza	16°33′ N, 16°14′ W	5324	Situated at 45 km from the Diama dam constructed on the Senegal River (low valley); commercial and agricultural centre; irrigated rice fields in the periphery; annual rainfall, 180–300 mm; mean annual temperature 28 °C (range, 24 °C [December–January] to 38 °C [May–July]); relative humidity, 42 % (June-July) to 88 % (August–September)
Boghé	Boghé, Brakna	16°35′ N, 14°16′ W	37,139	Located along the Senegalese-Mauritanian border and along the Senegal River (middle valley); commercial and agricultural centre; irrigated rice fields in the periphery; annual rainfall, 300–380 mm; mean annual temperature 35 °C (range, 28 °C [December–January] to 42 °C [April–June]); relative humidity, 26 % (May–June) to 72 % (August–September)
Gouraye	Selibabi, Guidimakha	14°54′ N, 12°27′ W	2009	Commercial and agricultural centre situated along the Senegalese-Mauritanian border (high valley); flood recession agriculture; annual rainfall, 300–480 mm; mean annual temperature 36 °C (range, 29 °C [December–February] to 43 °C [April–May]); relative humidity, 28 % (May–June) to 74 % (August–September)
Ghabou	Selibabi, Guidimakha	18°18′ N, 14°98′ W	4120	Located at the upstream sector of the Senegal River (intersection of 3 countries, i.e., Mauritania, Senegal, and Mali); water retention structure (called Kara Koro) for flood recession cropping; annual rainfall, 300–500 mm; mean annual temperature 36 °C (range, 29 °C [December–February] to 43 °C [April–May]); relative humidity, 28 % (May–June) to 76 % (August–September)
Sahelo-Saharan transition zone				
Kobeni	Kobeni, Hodh Elgharbi	15°49′ N, 09°24′ W	2336	Administrative, commercial, and agricultural-pastoral centre along Aioun-Nioro route that links Nouakchott (980 km northwest of Kobeni) and Mali; lies about 20 km from the border with Mali; city surrounded by several artificial lakes, ponds, and backwater; annual rainfall, 200–320 mm; mean annual temperature 35 °C (range, 28 °C [January–February] to 44 °C [April–May]); relative humidity, 19 % (May–June) to 76 % (August–September)
Tamcheket	Tamcheket, Hodh Elgarbi	17°14′ N, 10°40 W	1915	Large town on a sand dune near a semi-permanent artificial lake that divides the town into northern (Tamcheket) and southern sector (Loued); a commercial and cultural centre at the crossroads of ancient tracks that led to the Tagant plateau; annual rainfall, 100–220 mm; mean annual temperature 30 °C (range, 27 °C [December–January] to 42 °C [April–June]); relative humidity, 20 % (May–June) to 66 % (August–September)
Kankossa	Kankossa, Assaba	15°56′ N, 11°31′ W	10,470	Situated about 80 km southeast of the regional capital, Kiffa; a 30-km long permanent lake in the city allows flood-recession crop production, palm production, and market gardening; annual rainfall, 300–400 mm; mean annual temperature 33 °C (range, 31 °C [January–February] to 42 °C [May–June]); relative humidity, 32 % (May–June) to 78 % (August–September)
Nbeika	Moudjeria, Tagant	17°59′ N, 12°15′ W	1956	A small oasis village situated 15 km from Moudjeria city and 480 km to the east of Nouakchott; surrounded by rocky cliffs and mountains overlain by sand dunes; permanent artificial lakes allow market gardening in the village; although situated in the Saharan desert, its mountainous geography, location on the Tagant plateau (altitude, 225 m), and rainfall are characteristics of the sahelo-Saharan zone; annual rainfall, 100–140 mm; mean annual temperature 36 °C (range, 28 °C [December–February] to 42 °C [May–July]); relative humidity, 25 % (May–June) to 63 % (August–September)
Saharan zone				
Aoujeft	Aoujeft, Adrar	19°58’ N, 13°04’ W	3241	450 km from Nouakchott; despite its scarce vegetation, arid climate, and low annual rainfall (rainy season from August–October), the Adrar mountain ranges create a unique microclimate; oasis; annual rainfall, 50–80 mm; mean annual temperature 37 °C (range, 25 °C [December–January] to 44 °C [May–June]); relative humidity, 22 % (May–June) to 55 % (September–October)
Akjoujt	Akjoujt, Inchiri	19°45′ N, 14°23′ W	5870	A mining town located 265 km to the northwest of Nouakchott; very short rainy season (September); oasis; main livelihood, camel herding; annual rainfall, 20–80 mm; mean annual temperature 37 °C (range, 26 °C [December–February] to 45 °C [May–July]); relative humidity, 18 % (May–June) to 55 % (September–October)
Nouakchott^c^	Capital city	18°06′ N, 15°57′ W	800,000	A coastal city that occupies a surface area of 1,000 km^2^ and extends 25 km from north to south; almost one-third of the Mauritanian population resides in Nouakchott; market gardens in Darnaim and Teyarett districts; annual rainfall, 50–100 mm; mean annual temperature, 30 °C (range, 22 °C in December to 40 °C in June); relative humidity, 30 % (May–June) to 80 % (August–September)

^a^Provinces are locally called ‘moughataa’ (there are 54 moughataas in the country), and regions refer to ‘wilaya’ (there are 15 wilayas or regions in the country)

^b^Data from Ref. [10]

^c^The present study was conducted in Dar Naim and Teyarett districts in the northern part of Nouakchott where market gardening is largely practiced. A previous study conducted in Nouakchott has shown that, among nine districts, Dar Naim and Teyarett have the highest malaria prevalence among febrile patients consulting at two main hospitals in the capital city [3]

### Study design

A cross-sectional comparative study was implemented to meet the objectives of the present study. The survey was conducted for four weeks at each study site during the ‘cool’ dry season just after the rainy season (from the fourth week of November to the third week of December 2011), during the ‘hot’ dry season (June 2012), and during the rainy season (October 2013). The survey was designed as a representative three-stage cluster sample of households proportional to the size of the population of each study site. The most recent nationwide list of households compiled for the housing and population census in Mauritania was used for this study. At first stage, districts within the city or town were selected by simple random sampling. The same districts were surveyed over 3 years. At second stage, 20 households were randomly selected from the listing units within each district at each season. The random sample of these clusters was composed of 50–60 children aged between two and nine years of age. Children meeting the following inclusion criteria were invited to participate in the present survey: age between two and nine years, presence in the randomly selected household on the day of the survey, and resident in the household for at least 1 month. Parasite rates in two to nine years old age group had historically been used to determine the level of endemicity in Africa [11].

### Laboratory examinations

Finger-prick capillary blood was collected with the capillary pipettes provided in the kit for rapid diagnostic test (RDT) for malaria (SD Bioline malaria antigen P.f/P.v or SD Bioline malaria antigen P.f/PAN; Standard Diagnostics, Inc., Yongin, Republic of Korea). The RDT was performed to detect the presence of malaria parasites. These RDT use two monoclonal antibodies that are specific to *P. falciparum* histidine-rich protein-2 (HRP-2) and *P. vivax* lactate dehydrogenase (LDH) to differentiate between *P. falciparum* and *P. vivax* (for P.f/P.v RDT) or *P. falciparum*-specific HRP-2 and genus-specific plasmodial LDH (pLDH) (for P.f/PAN RDT) to differentiate between *P. falciparum* and non-*P. falciparum* species.

Fingerprick capillary blood was also used to prepare and examine Giemsa-stained thin and thick smears under the microscope, according to standard World Health Organization (WHO) procedures [12]. Blood films were examined at a magnification of 1000× to confirm the presence of malaria parasites detected by RDT, identify the *Plasmodium* species, and determine the parasite density. The number of asexual parasites was counted against 200 white blood cells (WBC) to calculate the parasite density, expressed as the number of asexual parasites per µl of blood. The number of asexual parasites was divided by the number of WBC and multiplied by an assumed WBC count of 8000 per µl.

All children with a positive smear and/or RDT result, including *Plasmodium ovale* and *Plasmodium malariae* infections, were immediately referred to the nearest health centre for antimalarial treatment. The importance of medical consultation was explained to the head of the household, and the field supervisor visited malaria-infected children in their homes the next day to ensure that appropriate treatment had been provided.

Quality control was performed by an experienced microscopist who re-examined 10 % of randomly selected positive and negative thick smears under the microscope. If less than 5 % of the results were discordant, the quality of microscopic examination was considered to be acceptable. If more than 5 % of the results were discordant, the laboratory technician was required to re-examine all smears, and a second quality control was performed until less than 5 % of the results were discordant.

### Mosquito collection and identification

Two methods were used for adult mosquito sampling [13]. First, indoor resting mosquitoes were sampled in the early morning by pyrethrum spray catch. Aerosol insecticide was used to knock down mosquitoes and to collect them on white cotton bed sheets spread on the floor. In each district, 10–15 bedrooms were randomly selected from the households included in the parasitological survey. Secondly, Centres for Disease Control (CDC) light traps were placed in the bedrooms of selected households next to a human volunteer under an untreated bed net. In each district, four light traps were operated each night for four weeks. After mosquito collection, mosquitoes were sorted in the field and identified using taxonomic keys for the morphological identification of adult mosquitoes [14, 15]. Further species identification within the *An. gambiae* species complex mosquitoes collected in 2013 was performed by PCR–RFLP at the Laboratoire d’Ecologie Vectorielle et Parasitaire in Dakar, Senegal [16].

### Statistical analysis

Aggregated parasite density by zones or years was expressed as the geometric mean and median. The comparisons of medians of parasite densities were performed with Mood’s median test and illustrated with violin plot. Proportions were compared using the Chi square test or Fisher’s exact test depending on the sample size. The level of statistical significance was set at *P* < 0.05. Statistical tests were performed using SigmaStat 3.5 (Systat Software, Inc., Point Richmond, CA) and R statistical language (R Foundation for Statistical Computing, Vienna, Austria).

### Ethical approval

The present study was reviewed and approved by the Mauritanian Ministry of Health, the only authority that provided ethical clearance in Mauritania in 2011. The purpose of the study was explained in local dialect to the heads of the selected households, who provided informed written consent on behalf of children between two and nine years of age living in the household.

## Results

A total of 3445 children between two and nine years of age were screened in 2011–2013 (Table 2). The mean age (± SD; range) of the enrolled children, 4.9 ± 2.4 (2–9) years, did not differ significantly (*P* > 0.05) in different epidemiological strata. The overall sex ratio (M:F) was 1.02, which was not significantly different in the three zones (*P* > 0.05). Of 3445 children, 143 (4.15 %) had malaria infection. Four human malaria species were observed in contrasting proportions during different seasons in three epidemiological strata: *P. falciparum* (n = 71; 2.06 %), *P. vivax* (n = 57; 1.65 %), mixed *P. falciparum*-*P. vivax* infections (n = 2; 0.06 %), *P. ovale* (n = 12; 0.35 %), and *P. malariae* (n = 1; 0.03 %).

**Table 2:** Characteristics of children screened for malaria infection during three different seasons in three epidemiological strata in Mauritania, 2011–2013

Characteristics	Sahelian zone	Sahelo-Saharan zone	Saharan zone
Number of children	1056	1330	1059
Age			
mean ± SD, range (year)	5.1 ± 2.3 (2–9)	5.0 ± 2.4 (2–9)	4.7 ± 2.4 (2–9)
2 to <5 years old (n, %)	434 (41.1)	530 (39.8)	544 (51.4)
5–9 years old (n, %)	622 (58.9)	800 (60.2)	515 (48.6)
Sex ratio (M/F)	0.96	1.0	1.1

### Seasonal occurrence of malaria

Analysis by epidemiological strata showed that the overall proportion of malaria-positive smears was significantly lower (*P* < 0.05) in the Sahelian zone (14 of 1056; 1.33 %), compared with that of Sahelo-Saharan (83 of 1330; 6.24 %) and Saharan zones (46 of 1059; 4.34 %) (Table 3). Malaria positivity rate did not differ significantly (*P* = 0.052) between Sahelo-Saharan and Saharan zones. During the ‘cool’ dry season in 2011, ‘hot’ dry season in 2012, and rainy season in 2013, there were 68 of 1181 (5.76 %), 3 of 1168 (0.26 %), and 72 of 1096 (6.57 %) children with positive smears, respectively. The difference in malaria positivity rate was statistically significant (*P* < 0.05) between the ‘hot’ dry season 2012 and ‘cool’ dry season 2011 or rainy season in 2013, but not between 2011 and 2013 (*P* = 0.473).

**Table 3:** Malaria-positive smears in 12 study sites in Mauritania

Zone/study sites	Subjects (n)	*Plasmodium species (n, %)*				All ***Plasmodium*** spp. (n, %)
		*P. falciparum*	*P. vivax*	*P. ovale*	*P. malariae*	
*Sahelian*	*1056*	*8 (0.76)*	*–*	*5 (0.47)*	*1 (0.09)*	*14 (1.33)*
2011	350	3 (0.86)	–	3 (0.86)	–	6 (1.71)
2012	369	–	–	–	–	–
2013	337	5 (1.48)	–	2 (0.59)	1 (0.30)	8 (2.37)
Keurmassen						
2011	50	1 (2.00)	**–**	**–**	**–**	1 (2.00)
2012	50	–	**–**	**–**	**–**	**–**
2013	117	1 (0.85)	**–**	**–**	**–**	1 (0.85)
Boghé						
2011	101	–	**–**	1 (0.99)	**–**	1 (0.99)
2012	101	–	**–**		**–**	**–**
2013	114	1 (0.88)	**–**	**–**	1 (0.88)	2 (1.75)
Gouraye						
2011	99	1 (1.01)	**–**	2 (2.02)	**–**	3 (3.03)
2012	99	–	**–**		**–**	**–**
2013	74	1 (1.35)	**–**	1 (1.35)	**–**	2 (2.70)
Ghabou						
2011	100	1 (1.00)	**–**	**–**	**–**	1 (1.00)
2012	119	**–**	**–**	**–**	**–**	**–**
2013	32	2 (6.25)	**–**	1 (3.12)	**–**	3 (9.38)
*Sahelo-Saharan*	*1330*	*62 (4.66)*	*16 (1.20)* ^a^	*5 (0.38)*	*–*	83 (6.24)*
2011	407	18 (4.42)	12 (2.95)	3 (0.74)	**–**	33 (8.11)
2012	451	**–**			**–**	**–**
2013	472	44 (9.32)	4 (0.85)	2 (0.42)	**–**	50 (10.59)
Kobeni						
2011	105	3 (2.86)	4 (3.81)	2 (1.90)	**–**	9 (8.57)
2012	111	**–**	**–**		**–**	**–**
2013	129	18 (13.95)	**–**	1 (0.78)	**–**	19 (14.73)
Tamcheket						
2011	102	2 (1.96)	6 (5.88)	1 (0.98)	**–**	9 (8.82)
2012	105	**–**	**–**		**–**	**–**
2013	105	5 (4.76)	4 (3.81)^a^	**–**	**–**	9 (8.57)
Kankossa						
2011	104	8 (7.69)	2 (1.92)	**–**	**–**	10 (9.62)
2012	109	**–**	**–**	**–**	**–**	**–**
2013	124	12 (9.68)	**–**	1 (0.81)	**–**	13 (10.48)
Nbeika						
2011	96	5 (5.21)	**–**	**–**	**–**	5 (5.21)
2012	126	**–**	**–**	**–**	**–**	**–**
2013	114	9 (7.89)	**–**	**–**	**–**	9 (7.89)
*Saharan*	*1059*	*1 (0.09)*	*43 (4.06)*	*2 (0.19)*	*–*	46 (4.34)*
2011	424	1 (0.24)	26 (6.13)		**–**	29 (6.84)
2012	348	**–**	3 (0.86)	2 (0.47)	**–**	3 (0.86)
2013	287	**–**	14 (4.88)	**–**	**–**	14 (4.88)
Teyarett^b^						
2011	109	1 (0.92)	15 (13.76)	2 (1.83)	**–**	18 (16.51)
2012	116	**–**	3 (2.59)		**–**	3 (2.59)
2013	109	**–**	8 (7.34)	**–**	**–**	8 (7.34)
Darnaim^b^						
2011	119	**–**	9 (7.56)	**–**	**–**	9 (7.56)
2012	125	**–**	**–**	**–**	**–**	**–**
2013	119	**–**	5 (4.20)	**–**	**–**	5 (4.20)
Aoujeft						
2011	146	**–**	2 (1.37)	**–**	**–**	2 (1.37)
2012	107	**–**	**–**	**–**	**–**	**–**
2013	59	**–**	1 (1.69)	**–**	**–**	1 (1.69)
Akjoujt						
2011	50	**–**	**–**	**–**	**–**	**–**
2012	ND					
2013	ND					

Overall data obtained in each zone, followed by data from each study site, are presented. The total numbers of malaria-positive individuals in each zone are in italics. Dash denotes n = 0. ND, not done. Asterisks (*) denote statistically significant difference (*P* < 0.05) in the proportions of malaria-positive smears between sahelian zone and both the sahelo-Saharan and Saharan zones. Malaria positivity rate between sahelo-Saharan and Saharan zones did not differ significantly (*P* = 0.052). The difference in malaria positivity rate was statistically significant (*P* < 0.05) between the ‘hot’ dry season 2012 (3 of 1168; 0.26 %) and ‘cool’ dry season 2011 (68 of 1181; 5.76 %) or rainy season in 2013 (72 of 1096; 6.57 %), but not between 2011 and 2013 (*P* = 0.473)

^a^Including two children with mixed *P. falciparum*-*P. vivax* infections

^b^Teyarett and Darnaim are districts in Nouakchott, the capital city of Mauritania

In the Sahelian zone, malaria-positive smears were found in 6 of 350 (1.71 %) children during the ‘cool’ dry season in 2011 and in 8 of 337 (2.37 %) children during the rainy season in 2013 (Table 3). During the same periods, there were higher proportions (*P* < 0.05) of malaria-positive smears in both the Sahelo-Saharan [33 of 407 children (8.11 %) and 50 of 472 children (10.59 %), respectively] and the Saharan zones [29 of 424 (6.84 %) and 14 of 287 (4.88 %), respectively]. The results obtained during the ‘hot’ dry season in 2012 showed either a very low malaria infection rate (3 of 348 subjects, 0.86 %; all 3 were due to *P. vivax* infection) in the Saharan zone or a total absence of detectable blood-stage malaria parasites in the Sahelian and Sahelo-Saharan zones, suggesting an apparent interruption of malaria transmission in these latter two zones during the ‘hot’ dry season.

#### *Plasmodium* species

The analysis of data by epidemiological strata showed that the Sahelian zone along the Senegal River was characterized by the lowest number of malaria-positive slides (14 of 1056 children, 1.33 %) during the study period (Table 3). There were eight children infected with *P. falciparum*, five with *P. ovale*, and one with *P. malariae*. *Plasmodium vivax* was not detected in the Sahelian zone. By comparison, the malaria burden in the sahelo-Saharan (83 of 1330 children, 6.24 %) and Saharan zones (46 of 1059 children, 4.34 %) were much higher (*P* < 0.05). In the Sahelo-Saharan zone, the majority of children were infected with *P. falciparum* (62 of 83 positive slides, 74.7 %), followed by *P. vivax* (14 of 83, 16.9 %), and *P. ovale* (five of 83, 6.0 %). Mixed *P. falciparum*-*P. vivax* infections (two of 83, 2.4 %) were also observed. Within the Sahelo-Saharan zone, most *P. falciparum* infections were observed near the border with Mali (*P* = 0.04) where war refugees have been resettled temporarily and where transmission was apparently interrupted during the hot season in 2012.

By contrast, in the Saharan zone, the majority of children were infected with *P. vivax* (43 of 46 positive slides, 93.5 %), and only one child (one of 46, 2.2 %) was infected with *P. falciparum*. In the Sahelo-Saharan and Saharan zones, *P. malariae* was not found in the present study.

### Parasite density

**Fig. 2:**
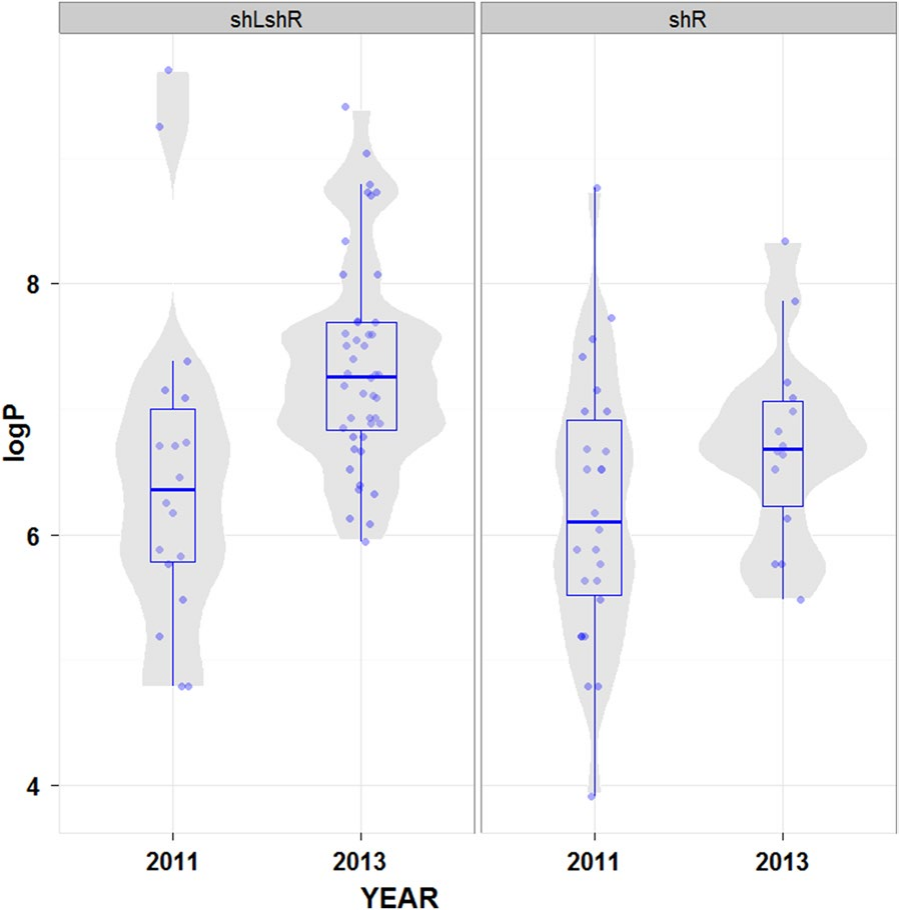
Distribution of *Plasmodium falciparum* and *Plasmodium vivax* parasite densities in Sahelo-Saharan and Saharan zones in Mauritania (2011 and 2013). Violin *plot* shows the probability density of parasitaemia. The superimposed *box plot* shows the median, interquartile range, and upper and lower extremes (*whiskers*). Each *circle* represents the logarithmic value of individual parasitaemia. shLshR, *P. falciparum* in the Sahelo-Saharan zone; shR, *P. vivax* in the Saharan zone. Mood’s comparison of medians was significant between 2011 and 2013 in the Sahelo-Saharan zone. Comparison of medians was not done in 2012 and in the Sahelian zone due to the small number of malaria-positive samples

The overall geometric mean parasite density [95 % confidence interval (95 % CI); range] and median of malaria-infected children (n = 143) were 804 asexual parasites/µl (671–963 asexual parasites/µl; 50–16,000 asexual parasites/µl) and 820 asexual parasites/µl, respectively. Parasite densities found in three epidemiological strata are summarized in Table 4. For *P. falciparum* infections in Sahelo-Saharan zone, the median parasite density in 2013 (1,420 asexual parasites/µl) was significantly higher (*P* = 0.018) than that observed in 2011 (580 asexual parasites/µl) (Fig. 2). Among 43 children infected with *P. vivax* in the Saharan zone, there was no statistically significant difference between the median parasite densities in 2011 (450 asexual parasites/µl) and 2013 (800 asexual parasites/µl) (*P* = 0.143).

**Table 4:** Parasite densities of *P. falciparum* and *P. vivax* infections in different epidemiological strata in Mauritania

Epidemiological strata	Number of isolates	Parasite density (asexual parasites/µl)		
		Geometric mean (95 % CI)	Range	Median
*P. falciparum*				
Sahelian	8	1280 (575–2840)	280–6400	1260
Sahelo-Saharan	62^a^	1220 (936–1600)	120–16,400	1200
Saharan	1	–	–	–
*P. vivax*				
Sahelian	0	–	–	–
Sahelo-Saharan	14^a^	273 (191–390)	80–820	300
Saharan	43	625 (459–852)	50–6400	680

Due to the small numbers of *P. ovale* and *P. malariae* infections, data on these *Plasmodium* species are not presented in the table. See text for findings on these species

^a^Excluding two children with mixed *P. falciparum*-*P. vivax* infections

### Entomological findings

A total of 3577 mosquitoes were captured in randomly selected households in the 12 surveillance sites in 2011–2013. The numbers of mosquitoes found in each study site during different seasons are summarized in Table 5. Based on the morphological features, there were 1014 (28.3 %) mosquitoes belonging to *Anopheles* spp. 1631 (45.6 %) *Culex* spp. and 932 (26.1 %) *Aedes* spp. *Anopheles* spp. (40 %) predominated over *Culex* (32 %) and *Aedes* (28 %) in the Sahelian zone during the ‘cool’ dry season in 2011. The proportions of *Anopheles* and *Culex* were similar (41.6 vs 43.9 %) during the 2013 rainy season in the Sahelo-Saharan transition zone. During the ‘hot’ dry season in 2012, the lowest number of mosquitoes was captured, and *Anopheles* spp. were absent in all study sites, except for Teyarett district in Nouakchott where *Anopheles gambiae* s.l. (n = 8) and *Anopheles pharoensis* (n = 1) were observed. In the two localities situated in northern Saharan zone, Akjoujt and Aoujeft, *Anopheles* mosquitoes were rarely captured during the entire study period.

**Table 5:** Mosquito abundance in three epidemiological strata in Mauritania during different seasons in 2011–2013

Zone/study sites	Number (%) of mosquitoes			
	*Anopheles* spp.	*Culex* spp.	*Aedes* spp.	All mosquitoes
*Sahelian zone*	*621*	*857*	*525*	*2003*
2011	430 (40.3)	338 (31.7)	299 (28.0)	1067
2012	0	14 (87.5)	2 (12.5)	16
2013	191 (20.8)	505 (54.9)	224 (24.4)	920
Keurmassen				
2011	0	12 (38.7)	19 (61.3)	31
2012	0	6 (100)	0	6
2013	23 (6.8)	230 (68.0)	85 (25.2)	338
Boghé				
2011	201 (41.1)	204 (41.7)	84 (17.2)	489
2012	0	8 (80.0)	2 (20.0)	10
2013	50 (17.0)	153 (51.9)	92 (31.2)	295
Gouraye				
2011	192 (44.0)	64 (14.7)	180 (41.3)	436
2012	0	0	0	0
2013	46 (37.4)	60 (48.8)	17 (13.8)	123
Ghabou				
2011	37 (33.3)	58 (52.2)	16 (14.4)	111
2012	0	0 (0)	0	0
2013	72 (43.9)	62 (37.8)	30 (18.3)	164
*Sahelo-Saharan zone*	*271*	*475*	*303*	*1049*
2011	12 (3.1)	170 (44.3)	202 (52.6)	384
2012	0	32 (74.4)	11 (25.6)	43
2013	259 (41.6)	273 (43.9)	90 (14.5)	622
Kobeni	0	109 (41.8)	152 (58.2)	261
2011	0	11 (100)	0	11
2012	106 (62.7)	54 (32.0)	9 (5.3)	169
2013				
Kankossa				
2011	9 (14.8)	28 (45.9)	24 (39.3)	61
2012	0	21 (65.6)	11 (34.4)	32
2013	97 (35.9)	127 (47.0)	46 (17.0)	270
Tamcheket				
2011	3 (13.0)	11 (47.8)	9 (39.1)	23
2012	0	0	0	0
2013	23 (24.2)	51 (53.7)	21 (22.1)	95
Nbeika				
2011	0	22 (56.4)	17 (43.6)	39
2012	0	0	0	0
2013	33 (37.5)	41 (46.6)	14 (15.9)	88
*Saharan zone*	*122*	*299*	*104*	*525*
2011	31 (28.2)	56 (50.9)	23 (20.9)	110
2012	9 (17.6)	31 (60.8)	11 (21.6)	51
2013	82 (22.5)	212 (58.2)	70 (19.2)	364
Teyarett, Nouakchott				
2011	22 (44.0)	19 (38.0)	9 (18.0)	50
2012	9 (39.1)	6 (26.1)	8 (34.8)	23
2013	58 (39.2)	42 (28.4)	48 (32.4)	148
Darnaim, Nouakchott				
2011	9 (31.0)	7 (24.1)	13 (44.8)	29
2012	0	23 (88.5)	3 (11.5)	26
2013	22 (23.9)	50 (54.4)	20 (21.7)	92
Akjoujt				
2011	0	6 (100)	0	6
2012	0	0	0	0
2013	0	39 (95.1)	2 (4.9)	41
Aoujeft				
2011	0	24 (96.0)	1 (4.0)	25
2012	0	2 (100)	0	2
2013	2 (2.4)	81 (97.6)	0	83

Overall data obtained in each zone, followed by data from each study site, are presented. The total numbers of mosquitoes in each zone are in italics. The collection periods correspond to the ‘cool’ dry season after the rainy season from the end of November to December 2011, ‘hot’ dry season in June 2012, and rainy season in October 2013

The *Anopheles* specimens collected during the study period consisted of at least six species (Table 6): *An. gambiae* s.l. (n = 610, 60.2 %), *Anopheles rufipes* (n = 238, 23.5 %), *An. pharoensis* (n = 134, 13.2 %), *Anopheles domicola* (n = 26, 2.6 %), *Anopheles ziemanni* (n = 5, 0.5 %), and *Anopheles rhodesiensis* (n = 1, 0.1 %). Boghé and Gouraye in the Sahelian zone and Kankossa and Kobeni in the Sahelo-Saharan zone recorded the highest number of *Anopheles* mosquitoes caught during the ‘cool’ dry season in 2011 and during the rainy season in 2013, respectively. Overall, mosquitoes from the *An. gambiae* s.l. complex were the most abundant and widely distributed. During the ‘cool’ dry season in 2011, *An. gambiae* s.l. mosquitoes were predominant in all three epidemiological strata. During the ‘hot’ dry season in 2012, *An. gambiae* s.l. mosquitoes were captured only in Teyarett district, Nouakchott. Molecular identification of the specimens collected during the 2013 rainy season revealed that *An. gambiae* sensu stricto and *An. arabiensis* occur in sympatry in most sites in the sahelo-Saharan and Saharan zones. In the Sahelian zone, *An. rufipes* appeared as the predominant species in indoor collections during the rainy season, followed by mosquitoes from the *An. gambiae* complex. *An. pharoensis* was also widespread in all three zones during the rainy season.

**Table 6:** *Anopheles* spp. identified at 12 surveillance sites during the ‘cool’ dry season in 2011 and rainy season in 2013

Sites	*Anopheles* species (n/N) (%)	
	2011	2013
Sahelian zone		
Keurmassen	0	*An. gambiae* s.l. 9/23 (39) *An. rufipes* 14/23 (61)
Boghé	*An. gambiae* s.l. 124/201 (62) *An. rufipes* 57/201 (28) *An. domicola* 20/201 (10)	*An. gambiae* s.l. 13/50 (26) *An. rufipes* 32/50 (64) *An. zemanni* 5/50 (10)
Gouraye	*An. gambiae* s.l. 132/192 (69) *An. rufipes* 54/192 (28) *An. domicola* 6/192 (3)	*An. rufipes* 35/46 (76) *An. pharoensis* 11/46 (24)
Ghabou	*An. gambiae* s.l. 37/37 (100)	*An. gambiae* s.l. 27/72 (38) *An. rufipes* 39/72 (54) *An. pharoensis* 6/72 (8)
Sahelo-Saharan zone		
Kobeni	0	*An. gambiae* s.s. 43/106 (41) *An. arabiensis* 38/106 (36) *An. pharoensis* 25/106 (24)
Kankossa	*An. gambiae* s.l. 8/9 (89) *An. rufipes* 1/9 (11)	*An. gambiae* s.l. 44/97 (45) *An. pharoensis* 53/97 (55)
Tamcheket	*An. gambiae* s.l. 3/3 (100)	*An. gambiae* s.s. 6/23 (26) *An. arabiensis* 11/23 (48) *An. rufipes* 6/23 (26)
Nbeika	0	*An. gambiae* s.s. 27/33 (82) *An. arabiensis* 4/33 (12) *An. pharoensis* 2/33 (6)
Saharan zone		
Teyarett, Nouakchott	*An. gambiae* s.l. 19/22 (86) *An. pharoensis* 3/22 (14)	*An. gambiae* s.s. 16/58 (28) *An. arabiensis* 23/58 (40) *An. pharoensis* 19/58 (33)
Darnaim, Nouakchott	*An. gambiae* s.l. 9/9 (100)	*An. arabiensis* 9/22 (41) *An. pharoensis* 13/22 (59)
Akjoujt	0	0
Aoujeft	0	*An. pharoensis* 1/2, (50) *An. rhodesiensis* 1/2 (50)

The collection period during the ‘cool’ dry season corresponds to the period from the last week of November to the third week of December in 2011. The collection period during the rainy season was in October 2013. During the ‘hot’ dry season in June 2012, only nine *Anopheles* mosquitoes (eight *An. gambiae s.l.* and 1 *An. pharoensis*) were captured in Teyarett district, Nouakchott. There was no *Anopheles* mosquito from the other 11 surveillance sites

*n* number of *Anopheles* species, *N* total number of *Anopheles* mosquitoes captured in the study sites

## Discussion

The present study is one of the most extensive epidemiological studies so far conducted in Mauritania. The survey covered 12 localities in nine administrative regions situated in three different epidemiological strata and included both parasitological and entomological data collection in 2011–2013. Despite the fact that the present survey was limited to children under nine years of age, previous studies on febrile patients spontaneously consulting at health centres have suggested that children aged between five and nine years represent the age group that is most affected by uncomplicated malaria and are the priority target population for malaria interventions [3, 7, 17]. Contrary to most previous studies conducted in Mauritania, in which symptomatic patients were enrolled in health centres and hospitals [2–5, 7, 8, 17, 18], the present study focused on children without signs and symptoms associated with malaria residing in randomly selected households. The survey was conducted for a period of four weeks in different seasons during three successive years in each of 12 sentinel sites.

In the Sahelian zone along the Senegal River, malaria burden in children two to nine years of age was relatively low, compared to other epidemiological strata, and was mostly due to *P. falciparum* monoinfections. Data reported in the present study from the Sahelian zone are in agreement with the results of a previous study in which health centres in Keurmassen and Boghé reported the lowest proportions of positive blood smears among febrile adult and paediatric patients during the rainy season (July–October 2010) and ‘cool’ dry season (December 2010–March 2011), as compared to febrile patients consulting health centres in Sahelo-Saharan (Kobeni, Kankossa, and Tamcheket) and Saharan (Nouakchott, Atar, and Akjoujt) zones [18]. Positive smears were observed mostly during the rainy season, and less than 1.7 % of the slides were positive during the rest of the year. As in the present study, most malaria-positive patients were infected with *P. falciparum* (32 of 39; 82 %), while seven of 39 patients had *P. ovale* or *P. malariae*. None of the febrile patients had *P. vivax*.

Malaria prevalence was also shown to be low in Rosso (16°30’N, 15°48’W), a city situated along the Senegal River, and its surrounding villages, in 2004–2006 [19]. In that study, 36 of 1431 (2.5 %) febrile paediatric and adult patients had positive smears, mostly due to *P. falciparum*, and 9 of 1040 (0.9 %) asymptomatic schoolchildren were malaria parasite carriers. The situation was similar on the other side of the border formed by the Senegal River, where malaria incidence has been low since the 1990s [20–22].

In the Sahelian zone along the Senegal River, transmission was seasonal, with an apparent absence of patent parasitaemia during the ‘hot’ dry season. These findings were somewhat unexpected because of long-standing a priori assumptions that the Mauritanian side of the Sahelian zone bordering Senegal was thought to be highly endemic for *P. falciparum* malaria. The low frequency of malaria-positive smears observed in the Sahelian zone may be at least partly attributed to an effective implementation of a set of intensive priority interventions in this zone over the past years with the support of Global Fund, including the distribution of long-lasting insecticide-impregnated nets (LLIN), intermittent preventive treatment during pregnancy, and prompt treatment with artemisinin-based combination therapy (ACT) [1]. During the study period 2011–2013, RDT, ACT, and LLIN were either not available or available in insufficient quantities in 2011 and 2012, partly due to the suspension of Global Fund, but they were available in sufficient quantities in 2013 in the public health sector in the Sahelian zone due to the aid provided by the Senegal River Development Organization (OMVS: Organisation pour la mise en valeur du fleuve Sénégal). Sulfadoxine-pyrimethamine was largely distributed and employed for intermittent preventive treatment in pregnant women in 2011–2013.

Malaria occurred more frequently in children in the Sahelo-Saharan transition zone, as compared to the Sahelian zone, during both the ‘cool’ dry season just after the rainy season in November–December 2011 as well as during the rainy season in October 2013. As in the Sahelian zone, there was no malaria-infected child in June 2012, suggesting an interruption of malaria transmission during the ‘hot’ dry season. In an earlier study in the Sahelo-Saharan zone, smear-positive febrile adult and paediatric patients (193 of 1271; 15.2 %) were mostly observed during the rainy season (July–October 2010), but smear-positive patients were also identified during the ‘cool’ dry season (December 2010–March 2011; 1.4–3.7 %) and hot dry season (April–June 2011; 0.5–1.7 %) [18]. The apparent absence of malaria in the Sahelo-Saharan zone during the hot dry season in 2012 is probably an incidental finding but would require further parasitological monitoring for confirmation. In the present study, *P. falciparum* monoinfection was predominant in the Sahelo-Saharan zone, but *P. vivax* monoinfection and *P. ovale* were responsible for close to one-fourth of malaria infections. Moreover, *P. falciparum*-*P. vivax* mixed infections (2 of 83; 2.4 %) were observed only in the transition zone. Similar proportions of different *Plasmodium* species (*P. falciparum*, 71.6 %; *P. vivax*, 21.6 %; *P. ovale*, 4.9 %; *P. malariae*, 0; mixed infections, 1.3 %) were reported among 222 malaria-infected febrile patients consulting health centres in Kobeni, Kankossa, and Tamchekett in 2010–2011 [18].

Nbeika and Tamcheket are situated near the same latitude as Nouakchott, and both have permanent or semi-permanent artificial lakes. Compared to Kobeni and Kankossa, two other study sites in the Sahelo-Saharan zone, the levels of malaria transmission were much lower in these two sites with a relatively small population of less than 2000 residents. The highest malaria transmission levels observed in this zone were during the rainy season in 2013 in Kobeni and Kankossa, both of which are situated near the border with Mali. These border areas, in particular Kobeni, are associated with considerable human migration (local nomad populations) and mass refugee flows. The mobility of parasite populations is facilitated in such circumstances, favouring the maintenance of malaria transmission or its re-emergence [23]. Human population movement remains a true challenge, but it is necessary to take into account such situations for an effective monitoring and control of malaria in the region.

In another recent study conducted in Kobeni and Timbedra (15°49′ N, 09°24 W; Hodh Echargui province) during the rainy season in September–October 2013 [24], the efficacy of artesunate-amodiaquine combination was assessed in *P. falciparum*-infected symptomatic patients of all ages. An adequate number of patients fulfilling the inclusion criteria (i.e., 65 patients) was recruited in less than three weeks, confirming the relatively high level of malaria transmission in these areas during the rainy season.

These findings in the Sahelo-Saharan zone are related with inadequate human resources, poor health infrastructure, and inadequate delivery of interventions, including insufficient supplies of RDT, ACT, sulfadoxine-pyrimethamine, and LLIN. Data on malaria interventions and quantities of essential drugs and preventive measures delivered to the zone are incomplete. However, available data from 2011–2013 strongly suggest that public health sector in the Sahelo-Saharan transition zone received much less quantities of intervention measures, as compared to the Sahelian zone. Preventive measures, such as LLIN and sulfadoxine-pyrimethamine for pregnant women, were not provided regularly, or even not provided at all to some study sites in the zone during the study period. In 2011 and 2012, RDT was either unavailable or its quantity was insufficient, but in 2013 the available quantity of RDT was sufficient to cover the needs in the Sahelo-Saharan zone. The quantity of ACT did not cover the needs of the population in the zone during the entire study period.

In the Saharan zone, malaria burden among children aged two to nine years old was lower than that of the Sahelo-Saharan transition zone but higher than that of the Sahelian zone. In sharp contrast to the Sahelian and Sahelo-Saharan zones, malaria transmission in the Saharan zone was characterized by a large predominance of *P. vivax*. Even during the dry season, there were children infected with *P. vivax* in Teyarett district in Nouakchott. These findings are in agreement with several studies conducted among febrile, symptomatic paediatric and adult patients presenting spontaneously at the Teyarett health centre, which showed an increasing incidence of *P. vivax* malaria and a decrease in *P. falciparum*-to-*P. vivax* proportions during recent years [4, 5, 8, 17, 18]. Moreover, one recent study has shown that *P. vivax* malaria occurs throughout the year among febrile residents consulting the Teyarett health centre and that the peak transmission period extends from October to December [8]. In agreement with the present findings, malaria incidence was higher in November (cool dry season) than in October (rainy season) in 2012 and 2013. Further entomological investigations have been engaged to describe more precisely vector mosquito population dynamics in the area and explore malaria transmission dynamics through the assessment of entomological inoculation rate and its distribution over time and space in Nouakchott. Other recent studies have shown that *P. vivax* malaria also occurs in febrile patients residing elsewhere in the capital city of Nouakchott, as in Darnaim district and several other districts [3]. These findings are related to a high concentration of Duffy-positive, *P. vivax*-susceptible human population, the presence of *Anopheles* mosquitoes, and man-made environmental modifications that favour the creation of larval habitats in Nouakchott [6, 9, 25]. These observations are also related to the complete absence of anti-malarial interventions in the Saharan zone, considered to be malaria-free until recent years. During the study period, no anti-malarial intervention was undertaken in 2011 and 2012 in the Saharan zone. It was only from 2013 that RDT, ACT, and preventive measures became available in limited quantities in Nouakchott.

The presence of *P. vivax* was confirmed in Nouakchott and Aoujeft in the present study. Malaria was not found in Akjoujt in the present study. The apparent absence of malaria transmission in Akjoujt is in agreement with the absence of *Anopheles* mosquitoes. However, in a year-long monitoring in Akjoujt health centre, 22 malaria-infected patients were found among 467 symptomatic patients spontaneously consulting the health centre [18]. In that study, 16 of 19 (84 %) and 3 of 3 (100 %) had *P. vivax* during the rainy season and ‘cool’ dry season, respectively. In Atar (20°31’N, 13°03’W), the regional capital of Adrar region that is located near but further north than Akjoujt and Aoujeft in the Saharan desert, *P. vivax*-infected symptomatic patients have been diagnosed using microscopy and rapid diagnostic test, confirmed using PCR, and successfully treated with chloroquine in 2013 [26]. The presence of *P. vivax* in Atar is at least partly related to frequent visits of some Nouakchott residents to Atar (about 500 km from the capital city), especially during the rainy season when some districts of the capital city are often flooded due to lack of water evacuation system. At present, available data suggest that *P. vivax* transmission in Aoujeft and Akjoujt appears to occur at a low level, possibly due to low human and *Anopheles* mosquito population densities. These data suggest that *P. vivax* could be more widely spread in Mauritania than currently known and possibly extend from the Sahelo-Saharan transition zone along the southern border with Mali (Kankossa, Kobeni) to as far north in the Saharan desert as Aoujeft, Akjoujt, and Atar, including the capital city, Nouakchott [4, 5, 8, 17, 18, 26].

In the present study, *P. vivax* was not found in the Sahelian zone along the Senegal River. In an earlier study performed in Rosso [19], *P. vivax* was diagnosed in seven symptomatic patients during the dry season in February 2004. However, the authors have suggested that it may be doubted whether local *P. vivax* transmission actually occurs in Rosso since the majority (5 of 7 patients) had a recent travel history to Nouakchott. Further surveys are warranted, particularly along the Senegal River, in the eastern regions, and oases in the northern regions to establish the current geographic limits of *P. vivax* transmission in Mauritania. The consequences of regional and seasonal human migratory movements, including nomads, migrants, and refugees fleeing armed conflicts in neighbouring countries, have not been evaluated in terms of health risks and may need to be taken into consideration to understand *P. vivax* distribution in the country.

The interruption of malaria transmission, as suggested by the complete absence of detectable blood-stage malaria parasites in the Sahelian and Sahelo-Saharan zones, is in agreement with the absence of *Anopheles* mosquitoes in these zones during the ‘hot’ dry season in 2012. Since the present survey was not conducted continuously in time, further surveillance is needed to determine monthly malaria incidence in these strata. In the Saharan zone, the low frequency of *P. vivax* malaria (3 of 348 children) observed in Teyarett, Nouakchott, but not in any other sentinel sites, during the dry season is compatible with a permanent presence of *Anopheles* mosquitoes in the capital city where numerous artificial larval habitats, including potable water leaks and standpipes, exist all year round [6, 8]. Nonetheless, this observation does not rule out relapse as primaquine is not used in the country [1, 27].

There is only one recent published entomological survey on the distribution of malaria vectors in Mauritania [28]. In that study, 21 localities in Trarza, Brakna, Assaba, Tagant, and Hodh Elgarbi regions, which are in the Sahelian and Sahelo-Saharan transition zones, were surveyed during the rainy season (October–November) in 2003. Three anopheline species were found: *An. gambiae* s.l., which was the most abundant species found in almost all localities, *An. pharoensis*, and *An. funestus*. Molecular analysis of the *An. gambiae* complex showed the predominance of *An. arabiensis*. Although mosquitoes were collected from different surveillance sites in the Sahelian and Sahelo-Saharan zones, the results of the previous study are in general agreement with those of the present study. However, more longitudinal studies are required to identify and characterize malaria vectors involved in *P. falciparum* and *P. vivax* transmission in different epidemiological strata in Mauritania.

The results presented in this study do not reflect the prevalence of malaria in the general population in Mauritania, nor do they intend to provide a detailed distribution map of its vector mosquitoes throughout the country. The target population consisting of children between two and nine years of age and discontinuous surveys during the three-year period are major limitations of the present study. However, the present study is one of the first steps towards a more comprehensive survey to map the current distribution of *Plasmodium* spp. and *Anopheles* spp. in different parts of the country and delimit malaria transmission zones with the aim to mobilize available resources and commit funds to appropriate areas for an effective malaria control programme.

## Conclusions

Although the malaria prevalence rate cannot be calculated from the data reported here, the present study provides some indications for further epidemiological work in the country. Moreover, the artificial division of the country into three epidemiological strata based on geographic and climatic features to analyse the current malaria situation provides a useful starting framework but should be complemented by taking into account other parameters related to human activities and cross-border as well as internal population movement.

The Sahelian zone along the Senegal River is characterized by relatively low malaria burden, compared to the other two epidemiological strata, due to malaria control interventions on both sides of the border with Senegal. To reduce *P. falciparum*-associated morbidity and mortality more effectively, more resources should be made available in the Sahelo-Saharan transition zone, where the problem of malaria is compounded by population movement (nomads, migrants, Nouakchott residents traveling to the zone) and the presence of war refugees. *P. vivax* malaria is a growing public health problem which is spreading in previously malaria-free zone in the Saharan desert, including the capital city where close to one-third of the country population is concentrated. Further studies are needed to define the geographic limits of *P. vivax*, especially in the northern and eastern parts of the country where surveys have rarely been conducted.

## Abbreviations

ACT: artemisinin-based combination therapy
CDC: Centres for Disease Control
LLIN: long-lasting insecticide-impregnated net
OMVS: Organisation pour la Mise en Valeur du fleuve Sénégal (Senegal River Development Organization)
RDT: rapid diagnostic test
WBC: white blood cells
WHO: World Health Organization

## References

[1] Malaria Control Unit. Mauritanian Ministry of Health. Plan stratégique de lutte contre le paludisme 2011–2015. Nouakchott; 2011.

[2] Cortes H, Morillas-Márquez F, Valero A (2003). Malaria in Mauritania: the first cases of malaria endemic to Nouakchott. Trop Med Int Health.

[3] Mint Lekweiry K, Ould Abdallahi M, Arnathau P, Trape JF (2009). Preliminary study of malaria incidence in Nouakchott, Mauritania. Malar J.

[4] Mint Lekweiry K, Basco LK, Ould Ahmedou Salem MS, Hafid JE, Marin-Jauffre A, Ould Weddih A (2011). Malaria prevalence and morbidity among children reporting at health facilities in Nouakchott, Mauritania. Trans R Soc Trop Med Hyg.

[5] Mint Lekweiry K, Ould Mohamed Salem Boukhary A, Gaillard T, Wurtz N, Bogreau H, Hafid JE (2012). Molecular surveillance of drug-resistant *Plasmodium vivax* using *pvdhfr*, *pvdhps* and *pvmdr1* markers in Nouakchott, Mauritania. J Antimicrob Chemother.

[6] Ould Ahmedou Salem MS, Mint Lekweiry K, Mint Hasni M, Konaté L, Briolant S, Faye O (2013). Characterization of anopheline (Diptera: Culicidae) larval habitats in Nouakchott, Mauritania. J Vector Borne Dis.

[7] Ould Ahmedou Salem MS, Ndiaye M, Ouldabdallahi M, Mint Lekweiry K, Bogreau H, Konaté L (2014). Polymorphism of the merozoite surface protein-1 block 2 region in *Plasmodium falciparum* isolates from Mauritania. Malar J.

[8] Ould Ahmedou Salem MS, Mint Lekweiry K, Mint Deida J, Ould Emouh A, Ould Weddady M, Ould Mohamed Salem Boukhary A (2015). Increasing prevalence of *Plasmodium vivax* among febrile patients in Nouakchott, Mauritania. Am J Trop Med Hyg.

[9] Mint Lekweiry K, Ould Ahmedou Salem MS, Basco LK, Briolant S, Hafid JE, Ould Mohamed Salem Boukhary A (2015). Malaria in Mauritania: retrospective and prospective overview. Malar J.

[10] United Nations Population Funds. Recensement Général de la Population et de l’Habitat. 2013. (http://countryoffice.unfpa.org/mauritania/2013/07/01/7177/rgph/).

[11] WHO. Terminology of malaria and of malaria eradication. Report of a Drafting Committee. Geneva: World Health Organization; 1963.

[12] WHO. Basic malaria microscopy. Learner’s guide. 2nd edn. Geneva: World Health Organization; 2010.

[13] WHO. Malaria entomology and vector control, Guide for participants. Geneva: World Health Organization; 2013.

[14] Gillies MT, de Meillon B (1968). The *Anophelinae* of Africa, South of the Sahara.

[15] Gillies MT, Coetzee MC (1987). A supplement to the *Anophelinae* of Africa South of the Sahara (Afrotropical region).

[16] Fanello C, Santolamazza F, della Torre A (2002). Simultaneous identification of species and molecular forms of the *Anopheles gambiae* complex by PCR-RFLP. Med Vet Entomol.

[17] Ould Ahmedou Salem MS, Basco LK, Ouldabdellahi M, Mint Lekweiry K, Konate L, Faye O (2015). Malaria-associated morbidity during the rainy season in Saharan and Sahelian zones in Mauritania. Acta Trop.

[18] Ouldabdallahi M, Ouldbezeid M, Ouldlemrabott MA, Ouldelvally A, Ouldkhairi ML, Ba MDD (2015). Etude de la morbidité et espèces de *Plasmodium* dans les différentes zones géo-climatiques de la Mauritanie. Bull Soc Pathol Exot.

[19] Ouldabdallahi M, Ouldbezeid M, Dieye M, Yacine B, Faye O (2011). Etude de la part du paludisme chez les consultants fébriles et des indices plasmodiques chez des écoliers dans la région du Trarza, République islamique de Mauritanie. Bull Soc Pathol Exot.

[20] Faye O, Fontenille D, Hervé JP, Diack PA, Diallo S, Mouchet J. Le paludisme en zone sahélienne du Sénégal. 1. Donnés entomologiques sur la transmission. Ann Soc Belg Med Trop. 1993;73:21–30. 8323405

[21] Faye O, Gaye O, Hervé JP, Diack PA, Diallo S (1993). Le paludisme en zone sahélienne du Sénégal. II. Indices parasitaires. Ann Soc Belg Med Trop.

[22] Faye O, Gaye O, Konaté L, Molez JF, Feller-Dansokho E, Hervé JP (1998). Prévision et prévention des épidémies de paludisme dans la vallée du fleuve Sénégal. Santé.

[23] Cohen JM, Smith DL, Cotter C, Ward A, Yamey G, Sabot OJ (2012). Malaria resurgence: a systematic review and assessment of its causes. Malar J.

[24] Ouldabdallahi M, Alew I, Ould Ahmedou Salem MS, Ba MDD, Ould Mohamed Salem Boukhary A, Ould Khairy ML (2014). Efficacy of artesunate-amodiaquine for the treatment of acute uncomplicated falciparum malaria in southern Mauritania. Malar J.

[25] Wurtz N, Mint Lekweiry K, Bogreau H, Pradines B, Rogier C, Ould Mohamed Salem Boukhary A (2011). Vivax malaria in Mauritania includes infection of a Duffy-negative individual. Malar J.

[26] Ould Ahmedou Salem MS, Ould Mohamed Lemine Y, Mint Deida J, Ould Lemrabott MA, Ouldabdallahi M (2015). Efficacy of chloroquine for the treatment of *Plasmodium vivax* in the Saharan zone in Mauritania. Malar J.

[27] WHO. World Malaria Report. Geneva: World Health Organization; 2014.

[28] Dia I, Ba H, Ould Mohamed SA, Diallo D, Lo B, Diallo M (2009). Distribution, host preference and infection rates of malaria vectors in Mauritania. Parasit Vectors..

